# Deployment-related trauma and post-traumatic stress disorder: does gender matter?

**DOI:** 10.1080/20008198.2018.1486123

**Published:** 2018-07-06

**Authors:** Christine Frank, Mark A. Zamorski, Jennifer E. C. Lee, Ian Colman

**Affiliations:** a School of Epidemiology, Public Health and Preventive Medicine, University of Ottawa, Ottawa, ON, Canada; b Military Personnel Research and Analysis, Department of National Defence, Ottawa, ON, Canada; c Directorate of Mental Health, Canadian Forces Health Services Group, Ottawa, ON, Canada; d Department of Family Medicine, University of Ottawa, Ottawa, ON, Canada

**Keywords:** Gender, post-traumatic stress disorder, PTSD, trauma, combat exposure, military, Género, Trastorno de Estrés Postraumático, TEPT, trauma, exposición a combate, militar, 性别, 创伤后应激障碍, PTSD, 创伤, 战斗暴露, 军事, • Women have higher odds of reporting PTSD compared to men.• Deployment-related trauma is positively linked with the odds of PTSD. Gender does not modify this link (i.e. no gender differences).• Interpersonal trauma is positively linked with the odds of reporting PTSD. Gender is a significant modifier of this association.• The interpersonal trauma–PTSD association is significantly stronger for women than for men.

## Abstract

**Objective:** Military research has attempted to identify whether women have an increased vulnerability to mental health issues following deployment-related trauma, but findings have been mixed. Most studies have controlled for childhood abuse, but not other non-deployment trauma (e.g. life-threatening illness), which may partly explain previous mixed results. This study assessed gender differences in the association between deployment-related trauma and post-traumatic stress disorder (PTSD) while controlling for non-deployment trauma.

**Methods:** Data came from the 2013 Canadian Forces Mental Health Survey. Regular or reserve personnel who had been deployed at least once were included in this study (*n* = 5980). Logistic regression was used to examine the interaction between gender and deployment-related trauma in predicting lifetime PTSD.

**Results:** After controlling for non-deployment trauma, the association of gender with PTSD went from being significant to being marginally significant. The interaction between gender and deployment-related trauma was not significant.

**Conclusion:** Though controlling for non-deployment trauma did not completely dissipate gender differences in PTSD, such differences were greatly reduced, indicating that these may be partly related to traumatic experiences outside deployment. As gender did not moderate the link between deployment-related trauma and PTSD, the findings suggest that trauma experienced while on deployment does not disproportionately affect women compared to their male counterparts.

## Abstract (es)

**Objetivo**: La investigación militar ha intentado identificar si las mujeres tienen una mayor vulnerabilidad a los problemas de salud mental después del trauma relacionado con el despliegue militar, pero los hallazgos han sido mixtos. La mayoría de los estudios han controlado el abuso infantil, pero no otros traumas no relacionados con el despliegue (p. Ej., Enfermedad potencialmente mortal), lo cual puede explicar parcialmente los resultados mixtos anteriores. Este estudio evaluó las diferencias de género en la asociación entre el trauma relacionado con el despliegue militar y el trastorno de estrés postraumático, al mismo tiempo que se controlaba los traumas no relacionados con despliegue militar.

**Métodos**: Los datos provienen de la Encuesta de Salud Mental de las Fuerzas Armadas Canadienses de 2013. El personal regular o de reserva que se había desplegado al menos una vez se incluyeron en este estudio (n = 5980). Se Utilizó regresión logística para examinar la interacción entre el género y el trauma relacionado con el despliegue en la predicción del TEPT a lo largo de la vida.

**Resultados**: Después de controlar por trauma no relacionado con el despliegue, la asociación de género con TEPT paso de ser significativa a solo marginalmente significativa. La interacción entre el género y el trauma relacionado con el despliegue no fue significativa.

**Conclusión**: Aunque el controlar por traumas no relacionados con el despliegue militar no disipó por completo las diferencias de género en el TEPT, tales diferencias se redujeron sustancialmente, lo que indica que pueden estar parcialmente relacionadas con experiencias traumáticas fuera del despliegue. Dado que el género no moderó la relación entre el trauma relacionado con el despliegue y el TEPT, los hallazgos sugieren que el trauma experimentado durante el despliegue no afecta en forma desproporcionada a las mujeres en comparación con sus pares masculinos.

## Abstract (zh)

目标：军事研究试图识别妇女在入伍相关的创伤后是否更容易受到精神健康问题的影响，但研究结果不一。大部分研究都控制了儿童期虐待，但没有控制其他与入伍无关的创伤（如，威胁生命的疾病），这可能部分解释了混合结果。 本研究评估了与部署相关的创伤和PTSD之间的关联中的性别差异，同时控制了非入伍相关创伤。

方法：数据来自2013年加拿大部队心理健康调查。 本研究纳入至少部署一次的定期或储备人员（n = 5980）。 Logistic回归用于检测性别和部署相关创伤之间在预测终身PTSD方面的相互作用。

结果：控制非入伍相关创伤后，性别与创伤后应激障碍的关联从显著变为边缘显著。 性别与入伍相关的创伤之间的相互作用并不显著。

结论：尽管控制非入伍相关创伤并未完全消除创伤后应激障碍中的性别差异，但这种差异大大减少，表明这可能与入伍以外的创伤性经历有部分关系。 由于性别并未降低部署相关创伤与创伤后应激障碍之间的联系，研究结果表明创伤的经历在入伍过程中不会影响女性比男性更为严重。

## 1. Introduction

### 1.1. Mental disorders in the military

Research suggests that post-traumatic stress disorder (PTSD) as well as other mental health issues are more prevalent in the military than in the general population (Goodwin et al., 2015; Kessler et al., 2014; McGuire et al., 2015; Rusu, Zamorski, Boulos, & Garber, 2016; Weeks, Zamorski, Rusu, & Colman, 2017). Mental health issues can be particularly deleterious in a military context, as they impact both individual and organizational outcomes, such as attrition (Hoge, Auchterlonie, & Milliken, 2006), lost work-days (Hoge, Terhakopian, Castro, Messer, & Engel, 2007), reduced productivity (Eibner, Ringel, Kilmer, Pacula, & Diaz, 2008), and decreased quality of life (Schurr, Lunny, Bovin, & Marx, 2009).

### 1.2. Deployment and mental disorders

Research has suggested a dose–response relation between deployment-related trauma and the subsequent risk of mental health issues (Dohrenwend, Turner, Turse, & Adams, 2006; Hoge et al., 2004). In the past 15 years, rates of deployment have increased, with more than 40,000 Canadian Forces personnel having been deployed in support of the mission in Afghanistan (Zamorski & Boulos, 2014). Of those deployed in support of that mission, approximately 13.5% had a diagnosed mental disorder attributed to the deployment (Boulos & Zamorski, 2013), with PTSD as the most common diagnosis (8%).

### 1.3. Gender differences in deployment-related PTSD

Not all who are exposed to a traumatic event develop PTSD, indicating there may be vulnerability factors that contribute to the development of PTSD (Yehuda, 1999). Given this, in recent years, research has sought to identify pre-enlistment characteristics that may predict an increased vulnerability to trauma. Most notably, gender has been explored as a potential predictor of increased PTSD following combat exposure; however, findings have been mixed. Some studies report no gender differences (e.g. Jacobson, Donoho, Crum-Cianflone, & Maguen, 2015; Maguen, Luxton, Skopp, & Madden, 2012), others report an increased risk of PTSD among women (e.g. LeardMann, Smith, Smith, Wells, & Ryan, 2009; Luxton, Skopp, & Maguen, 2010; Mustillo & Kysar-Moon, 2016; Xue et al., 2015), and one study reported an increased risk of PTSD among men (i.e. Hourani, Willians, Bray, Wilk, & Hoge, 2016). The authors of a meta-analysis on gender as a risk factor for PTSD suggested that it is important to control for pre-deployment factors in order to accurately assess gender differences in future research (Crum-Cianflone & Jacobson, 2014).

One potential explanation for the mixed results may be that few studies have controlled for other non-deployment-related traumatic experiences (i.e. trauma experienced outside deployment) and, in particular, past sexual trauma. Some researchers have explored the role of childhood adversity (e.g. physical abuse) as a determinant of mental health among military personnel (Cabrera, Hoge, Bliese, Castro, & Messer, 2007; Lee, Phinney, Watkins, & Zamorski, 2016; Seifert, Polusny, & Murdoch, 2011), but they have largely ignored the potential effects of other lifetime traumatic events. This is problematic, as prior trauma exposure is a significant predictor of PTSD among military personnel (Xue et al., 2015), and women may be particularly vulnerable to specific types of trauma.

It has been hypothesized that the disproportionate vulnerability to trauma in women is due to the nature of the traumatic events that women experience (DePrince & Freyd, 2002). Betrayal trauma theory suggests that not all negative events are equally traumatic and that women report higher rates of PTSD than men because they are more likely to experience medium- or high-betrayal traumatic events, which are more likely to lead to PTSD (DePrince & Freyd, 2002). Examples of high-betrayal trauma are sexual, emotional, or physical abuse perpetrated by someone close to the victim (e.g. a romantic partner or friend). Medium-betrayal traumatic events are these same traumatic experiences perpetrated by someone not close to the victim (e.g. an acquaintance or a stranger). Low-betrayal traumatic events are experiences that are not interpersonal in nature, such as natural disasters or car accidents. Research supports this supposition, showing that women are more likely to experience interpersonal (or medium-/high-betrayal) traumas, such as sexual assault or domestic violence, while men are more likely to experience non-interpersonal (or low-betrayal) trauma, such as being in an automobile accident or witnessing death or injury (Ghafoori, Barragan, & Palinkas, 2013; Tolin & Foa, 2008). In addition, one study found that high-betrayal trauma mediated the link between gender and PTSD (Tang & Freyd, 2012). Interpersonal trauma, such as sexual, emotional, or physical abuse perpetrated by another person, has especially been believed to result in a loss of trust in the world (Ratcliffe, Ruddel, & Smith, 2014). Along with the fact that women have been found to hold more negative views of themselves (Simmon & Granvold, 2005), this may contribute to important changes in how women come to see the world after interpersonal traumatic experiences that subsequently impact the severity and persistence of their PTSD symptoms. Given this potential vulnerability among women, it is important to control for non-deployment-related traumatic experiences in order to fully assess gender differences in PTSD following deployment-related trauma.

### 1.4. Relevance

These findings are particularly important given the growing role of women in the military. In Canada, 14.8% of Regular Force and Primary Reserve personnel are women (Department of National Defence, 2014). In the United States (US), recent deployments in support of the missions in Iraq and Afghanistan have included more women than all other conflicts in US military history (Street, Vogt, & Dutra, 2009). Although women have been permitted in combat roles in the Canadian Armed Forces (CAF) since 1989 (Department of National Defence, 2014), there have recently been changes to restrictions in the US military, whereby women will now be integrated into combat roles. Thus, it is important to accurately identify whether women are at increased risk of developing PTSD following deployment-related traumatic experiences and to understand the mechanisms that may account for any increased risk.

### 1.5. Current study

Although there is a growing amount of literature on gender differences in the prevalence of mental health outcomes in previously deployed military personnel, currently no work has been done to examine gender differences while controlling for past traumatic experiences beyond child abuse victimization. With the increase of women in the military and the expansion of women’s roles, it is important to understand gender-based risk differences in PTSD following deployment. The current study has two objectives: the main objective is to assess whether gender modifies the association between deployment-related trauma and PTSD; the secondary objective is to assess gender differences in the link between non-deployment-related trauma and PTSD and, in particular, whether women are more vulnerable when the traumatic event is interpersonal (i.e. medium or high betrayal) compared to when it is not interpersonal (i.e. low betrayal).

## 2. Method

### 2.1. Data source

Data were collected as part of the 2013 Canadian Forces Mental Health Survey (CFMHS) (Statistics Canada, 2014). The target population for the 2013 CFMHS was all full-time Regular Force members of the CAF, as well as Reservists who have been deployed in support of the mission in Afghanistan since 2001. The sample frame for the CFMHS survey was an administrative list of all Regular Force and all eligible Reserve Force members as of September 2012, which was provided to Statistics Canada by the Department of National Defence. The sample was selected using a stratified random sampling approach. Statistics Canada interviewers collected the data using computer-assisted, face-to-face interviews between April and August 2013. The 2013 CFMHS has an excellent response rate of approximately 80% for Regular Force and 79% for Reserve Force members. Because the primary goal of this paper is to explore how gender influences the relationship between deployment-related trauma and mental health problems, only those who had been deployed at least once (and hence had an opportunity for deployment-related trauma exposure) were included in this sample (Regular Force *n* = 4580; Reserve Force *n *= 1460).

### 2.2. Measures

#### 2.2.1. Socio-demographic characteristics

Socio-demographic variables that were examined included gender, age, ethnic origin (white or non-white), marital status (single, separated/divorced/widowed, or married/common-law), income adequacy (whether respondents have difficulty meeting basic expenses such as food, shelter, and clothing; yes/no), and highest educational attainment (less than secondary, secondary, some post-secondary, and diploma or degree).

#### 2.2.2. Post-traumatic stress disorder

The World Health Organization Composite International Diagnostic Interview (WHO-CIDI) (Kessler & Ustun, 2004) was used to assess the presence of lifetime PTSD. The WHO-CIDI assesses disorders according to the criteria in the Diagnostic and Statistical Manual of Mental Disorders (DSM) used by the American Psychiatric Association.

#### 2.2.3. Deployment-related trauma

Traumatic experiences while on deployment were measured using an eight-item questionnaire, derived from the larger 34-item Combat Exposure Scale (Hoge et al., 2004; Killgore et al., 2008), which asked participants to indicate whether they had ever experienced the indicated scenario on any deployment. Items were meant to capture a wide array of combat-related traumatic experiences. The eight-item subscale has been found to have the same model fit as the full scale, and was highly correlated with the full scale (*r* = .85, *p* < .001) (R. Nesdole, personal communication, 22 March 2017) (see Table 1 for additional detail on the items).

**Table 1.** Trauma rates by gender.

Type of trauma	Men, % [95% CI]	Women, % [95% CI]
Deployment-related trauma		
Known someone seriously injured or killed	5.5% [4.0; 6.9]	3.7% [0.0; 8.0]
In threatening situation	30.1% [28.8; 31.3]	14.0% [11.1; 16.9]
Ever been injured	24.2% [23.0; 25.4]	17.7% [14.6; 20.8]
Seen ill or injured women or children who you were unable to help	39.8% [38.4; 41.2]	32.5% [28.4; 36.6]
Ever received incoming artillery, rocket, or mortar fire	58.7% [57.3; 60.2]	48.1% [43.6; 52.7]
Ever felt responsible for the death of Canadian or ally	6.0% [5.4; 6.7]	5.4% [3.7; 7.0]
Had a close call (e.g. shot but protective gear saved you)	23.4% [22.2; 24.6]	8.6% [6.1; 11.2]
Had difficulty distinguishing between combatants and non-combatants	34.5% [33.1; 35.9]	14.4% [11.7; 17.2]
Non-deployment-related trauma		
*Non-interpersonal trauma*		
Civilian in a war zone	5.5% [3.1; 7.1]	3.7% [2.1; 5.4]
Civilian in a region of terror	4.4% [3.8; 5.0]	3.7% [2.1; 5.3]
Refugee	0.7% [0.4; 0.9]	–^ a^
Automobile accident	28.2% [26.9; 29.5]	19.3% [18.9; 19.6]
Life-threatening illness	7.3% [6.5; 8.1]	5.4% [3.4; 7.3]
Unexpected death of loved one	45.2% [43.7; 46.6]	43.4% [39.3; 47.6]
Child’s serious illness	6.1% [5.4; 6.8]	5.3% [3.5; 7.1]
Witnessed physical fights at home	11.8% [10.8; 12.7]	16.4% [13.3; 19.5]
Traumatic event to loved one	18.4% [17.3; 19.6]	17.2% [13.9; 20.6]
*Interpersonal* *trauma*		
Beaten up as a child by caregiver	6.9% [6.1; 7.6]	9.4% [6.9; 12.0]
Beaten up by spouse or romantic partner	1.1% [0.8; 1.4]	9.1% [6.3; 11.8]
Beaten up by somebody else	1.6% [0.6; 2.7]	5.3% [3.4; 7.3]
Sexual assault	2.5% [2.1; 3.0]	24.6% [21.1; 28.1]
Unwanted sexual touching	6.2% [5.4; 6.9]	42.6% [38.3; 46.9]
Stalked	5.8% [5.1; 6.5]	18.9% [15.5; 22.2]

^a ^The number of women who reported being a refugee was too small to report, as per Statistics Canada protocol on releasibility of data.

CI, confidence interval.

#### 2.2.4. Non-deployment-related trauma

Non-military trauma exposure was assessed using the 28-item inventory from the PTSD module of the WHO-CIDI version 3.0. First, three items that denoted deployment-related trauma were removed (e.g. have you ever participated in combat, either as a member of the military, or as a member of an organized non-military group?), given their overlap with the more specific deployment-related items listed in Table 1. Next, two independent researchers went through the list of remaining items and noted whether or not the remaining experiences would be likely to occur while on deployment. Then, the lists were compared and only items for which there was agreement that the experiences would not occur while on deployment were retained. Consequently, 10 items denoting experiences that could also occur (both conceptually and empirically) while on deployment were removed from the scale. Witnessing a human-made disaster, being held up with a weapon, and accidentally injuring others were examples of traumatic events that may have happened to military personnel while on deployment or pre-/post-deployment.

The final 13 items (Table 1) were then categorized as either interpersonal trauma (those that would be considered medium or high betrayal) or non-interpersonal trauma (those that would be considered low betrayal). Item categorization was strongly based on a factor analysis conducted on the full scale (Bennet & Zamorski, 2015).

### 2.3. Analysis

To assess our study objectives, a block-wise logistic regression was conducted using Stata version 13.1 with lifetime PTSD as the outcome. The analysis was conducted using survey weights generated by Statistics Canada for each survey. Weights provided by Statistics Canada capture the complex sampling scheme and non-response adjustments and were applied for all analyses, making them representative of the source population. Variance was estimated using bootstrap methods with 500 replicate weights also provided by Statistics Canada.

The first model included only gender, deployment-related trauma, and their two-way interaction term. To assess whether the mixed results are in part due to past studies failing to control for past trauma, we first need to assess whether there are gender differences in the association between military trauma and PTSD, and whether those differences attenuate or disappear after controlling for non-deployment-related trauma. In the second model, all socio-demographic variables were added (age, marital status, income adequacy, education, and ethnicity). The third model added interpersonal trauma and non-interpersonal trauma and their two-way interaction terms with gender.

Owing to small cell sizes in some groups (e.g. few men reported four or more interpersonal trauma experiences and few women experienced more than four deployment trauma experiences), we treated trauma exposure as categorical variables reflecting low, medium, and high exposure to traumatic events based on approximate tertiles of exposure. For deployment-related trauma, those who reported none or one traumatic event were categorized as having experienced low trauma, those who reported two or three unique traumatic events were categorized as having experienced moderate trauma, and those who reported four or more unique traumatic events were categorized as having experienced high trauma. For interpersonal and non-interpersonal trauma, the number of traumatic events reported was used to categorize respondents as either low trauma (none or one event), moderate trauma (two events), or high trauma (three or more events). In sensitivity analyses, we found no meaningful differences in terms of outcome prevalence in those zero versus one trauma types, justifying their combination into a single category. In addition, in sensitivity analyses, the pattern of findings was coherent when trauma variables were treated as continuous scales.

To explore more sensitively the possibility of interactions between gender and trauma, the *margins* command in Stata (Long & Freese, 2014) was used to assess whether there were statistically significant differences between genders and to compare the adjusted predicted probabilities across groups. The *margins* command can provide predictive margins (i.e. the probability of the outcomes given specified values for gender and trauma exposure), average marginal effects (i.e. the difference in the probability of the outcome between genders while holding covariates constant), and contrasts of average marginal effects (i.e. whether the average marginal effects significantly differ between genders).

## 3. Results

The prevalence of trauma exposure as a function of gender is displayed in Table 1. Men were more likely to experience all but two deployment-related traumas (i.e. known someone seriously injured or killed; ever felt responsible for the death of Canadian or ally). For non-interpersonal traumas, the only gender difference found involved men being more likely to experience automobile accidents. Women were more likely to experience all interpersonal-related traumas, with the exception of being beaten up as a child by a caregiver.

We examined socio-demographic and military characteristics by gender. Using the 95% confidence intervals (CIs) as a guide, substantial differences between the genders were noted. From Table 2, it can be seen that women were more likely to be single or widowed, separated, or divorced and less likely to be married than men. Women were also more likely to have a diploma or degree and less likely to have less than secondary or secondary education. Last, women were more likely to be a officers and less likely to be junior rank.

**Table 2.** Prevalence of socio-demographic characteristics among military personnel.

	Men (88.89%)	Women (11.11%)
Characteristics	% [95% CI]	% [95% CI]
Age group (years)		
< 25	4.7 [3.9; 5.4]	2.1 [0.5; 3.6]
25–34	34.0 [32.6; 35.7]	31.2 [27.2; 35.1]
35–44	33.1 [31.8; 34.5]	38.5 [34.6; 42.4]
> 44	28.2 [27.1; 29.4]	28.3 [24.4; 32.2]
Ethnicity		
White	92.3 [91.5; 93.0]	91.4 [89.0; 93.8]
Non-white	7.7 [7.0; 8.5]	8.6 [6.3; 11.0]
Marital status		
Single	18.0 [16.9; 19.1]	22.5 [18.9; 26.0]
Married/common-law	74.2 [73.0; 75.5]	65.3 [61.2; 69.4]
Widowed/separated/divorced	7.8 [7.0; 8.5]	12.2 [9.3; 15.2]
Difficulty meeting expenses	6.1 [5.4; 6.8]	6.2 [3.8; 8.5]
Highest education attained		
Less than secondary	4.4 [3.8; 4.9]	1.2 [0.4; 2.1]
Secondary	28.1 [26.8; 29.4]	18.4 [15.1; 21.8]
Some post-secondary	9.2 [8.3; 10.0]	7.8 [5.4; 10.1]
Diploma or degree	58.4 [57.0; 59.7]	72.5 [68.8; 76.3]
Rank		
Junior	46.9 [46.5; 47.3]	42.2 [41.8; 42.6]
Senior	31.9 [31.6; 32.1]	28.7 [28.4; 29.0]
Officer	21.2 [20.9; 21.6]	29.1 [28.8; 29.4]
Element		
Navy	16.5 [15.4; 17.6]	13.2 [10.3; 16.2]
Army	59.9 [58.4; 61.3]	58.6 [54.5; 62.8]
Air Force	23.6 [22.4; 24.9]	28.1 [24.0; 32.3]

CI, confidence interval.

Preliminary results pointed to a significant effect of gender, where women were over four times more likely to report PTSD than men, as well as an effect of deployment-related trauma, where an increase in trauma exposure (e.g. low to moderate) resulted in an almost three-fold increase in the probability of PTSD. The interaction between gender and deployment-related trauma in predicting PTSD was not significant (see Models 1 and 2 in Table 3 for results).

**Table 3.** Effects of gender and trauma on post-traumatic stress disorder.

	Model 1			Model 2^a^			Model 3^a^		
Predictor	OR	95% CI	*p*	OR	95% CI	*p*	OR	95% CI	*p*
Gender	4.58	[2.07; 10.14]	< .001	4.49	[1.59; 12.67]	.005	2.82	[0.96; 8.33]	.06
Deployment-related trauma	3.08	[2.62; 3.62]	< .001	2.87	[2.34; 3.52]	< .001	2.58	[2.20; 3.01]	< .001
Gender × Deployment trauma	0.80	[0.58; 1.12]	.19	0.81	[0.52; 1.24]	.33	0.80	[0.57; 1.12]	.19
Interpersonal trauma	–	–	–	–	–	–	1.76	[1.48; 2.08]	< .001
Gender × Interpersonal trauma	–	–	–	–	–	–	1.47	[1.00; 2.17]	.05
Non-interpersonal trauma	–	–	–	–	–	–	1.63	[1.41; 1.88]	< .001
Gender × Non-interpersonal trauma	–	–	–	–	–	–	0.80	[0.53; 1.21]	.30

^a^Adjusted for socio-demographic characteristics.

OR, odds ratio; CI, confidence interval.

**Figure 1.**
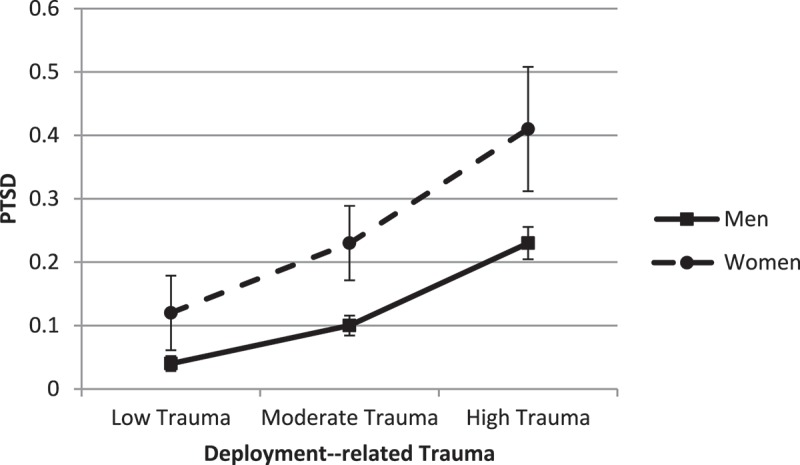
Predicted probability of post-traumatic stress disorder (PTSD) across deployment-related trauma.

We further explored gender differences in the association between deployment-related trauma and PTSD without controlling for non-deployment-related trauma. As expected, there were no gender differences in the strength of relationship between deployment-related trauma and PTSD (difference = 0.04, *SE* = 0.03, *t *= 1.21, *p* = .23, 95% CI −0.02; 0.01). Both genders reported an increase in the probability of PTSD as exposure to deployment-related trauma increased. Women reported a significantly higher probability of PTSD at low, moderate, and high levels of trauma (all *ps* < .005) (Figure 1).

After controlling for past trauma, there was a substantial decrease in the effect of gender, with gender now being only a marginally significant predictor of PTSD. Women were almost three times more likely to report PTSD than men. There was a significant effect of all three types of trauma (i.e. deployment-related trauma, interpersonal trauma, and non-interpersonal trauma), with the likelihood of developing PTSD increasing as trauma exposure increased. Only the interaction between gender and interpersonal trauma was significant (see Model 3 in Table 3 for results).

**Figure 2.**
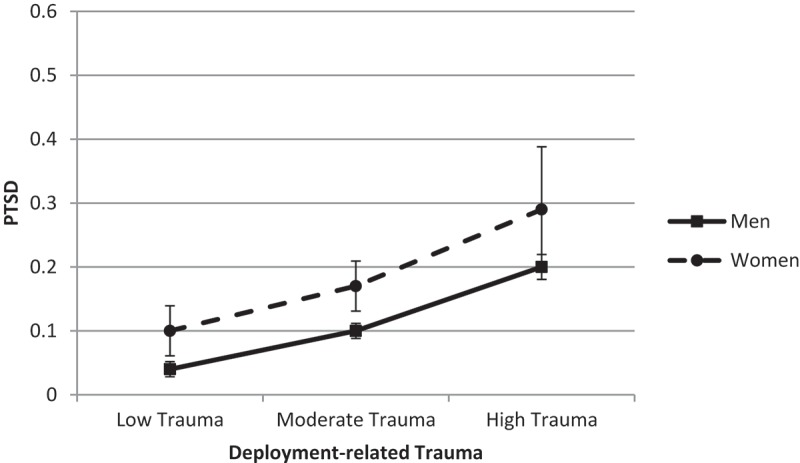
Predicted probability of post-traumatic stress disorder (PTSD) across deployment-related trauma, controlling for past trauma.

Again, we explored gender differences in the association between deployment-related trauma and PTSD, while controlling for non-deployment-related trauma. The association between deployment-related trauma and PTSD did not differ by gender (difference = 0.01, *SE* = 0.03, *t* = 0.81, *p* = .81, 95% CI −0.04; 0.06). Still, women reported a significantly higher likelihood of reporting PTSD than men when exposed to low or moderate trauma levels. Women were only marginally more likely to report PTSD than men when trauma was high (Figure 2).

**Figure 3.**
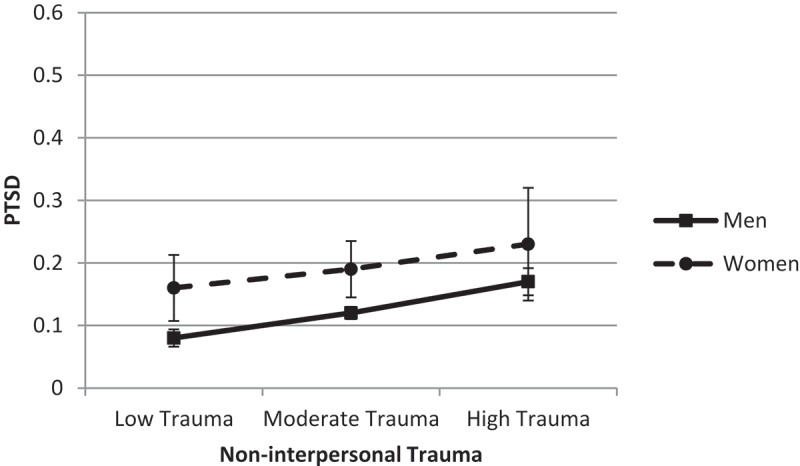
Predicted probability of post-traumatic stress disorder (PTSD) across non-interpersonal trauma.

We also looked at gender differences in the association between non-interpersonal trauma and PTSD. The strength of the association between non-interpersonal trauma and PTSD did not differ by gender (difference = − 0.01, *SE* = 0.03, *t* = − 0.37, *p* = .71, 95% CI −0.07; 0.04) (Figure 3). Again, women had a significantly higher probability of reporting PTSD when exposure to trauma was low or moderate (both *p* < .01), but not when exposure was high (*p* = .23).

**Figure 4.**
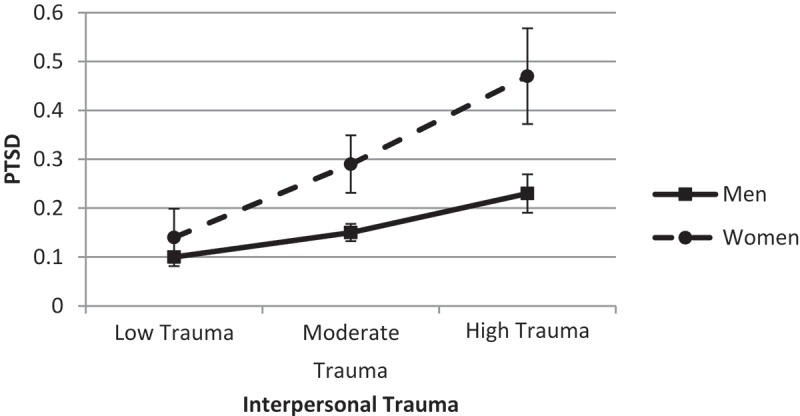
Predicted probability of post-traumatic stress disorder (PTSD) across interpersonal trauma.

Last, we explored the significant gender by interpersonal trauma interaction. Results showed that the link between interpersonal trauma and PTSD differed significantly by gender (difference = 0.07, *SE* = 0.02, *t* = 3.63, *p* < .001, 95% CI 0.03; 0.11). The association between interpersonal trauma and PTSD was stronger among women (*B* = 0.13, *SE* = 0.02, *t* = 6.89, *p* < .001, 95% CI 0.09; 0.16) relative to men (*B* = 0.05, *SE* = 0.01, *t* = 6.17, *p* < .001, 95% CI 0.09; 0.16). There was no significant difference between men and women when trauma exposure was low (*p* = .13), but the genders significantly differed when trauma exposure was moderate or high (both *p* < .001) (Figure 4).

## 4. Discussion

The primary purpose of this study was to assess gender differences in PTSD following deployment-related trauma while controlling for non-deployment-related trauma exposure (i.e. interpersonal trauma and non-interpersonal trauma). Gender did not modify the association between deployment-related trauma and PTSD in any of the analyses, suggesting that women do not have a disproportionate risk of deployment-related PTSD. However, the results suggest that women have an overall increased risk of PTSD. In line with some past research, when the model did not include non-deployment-related trauma, there was a significant effect of gender, where women were more likely to report PTSD (Luxton et al., 2010; Mustillo & Kysar-Moon, 2016; Xue et al., 2015). However, after controlling for non-deployment-related trauma, the gender differences decreased considerably, indicating that non-deployment trauma exposure substantially contributes to gender differences in PTSD. In support of this, there were significant gender differences among those with little to no exposure to deployment-related trauma. Accordingly, past research has found that female military personnel have higher rates of PTSD than male military personnel before having been deployed (Aggarwal, n.d.). Some researchers have argued that, rather than differences in trauma exposure, differences in cognitive factors, such as trauma memory records or self-schemas, contribute to a higher prevalence of PTSD among women, as these may affect the manner in which individuals process, recall, and respond to traumatic experiences (Simmons & Granvold, 2005). Indeed, there is evidence that gender differences in acute responses to trauma contribute to subsequent gender differences in PTSD (Irish et al., 2011). As such, women would experience higher levels of PTSD regardless of the number of traumatic experiences they had, resulting in a generalized increased risk of developing PTSD, rather than an increasing vulnerability as the number of traumatic experiences increases.

The secondary objective of the paper was to identify whether women were more vulnerable to interpersonal trauma (i.e. medium or high betrayal) compared to non-interpersonal trauma (i.e. low betrayal). The results proffered support for the betrayal theory. First, a substantially higher proportion of women reported experiencing interpersonal trauma events compared to men, indicating that women are indeed at a higher risk than men of experiencing medium- or high-betrayal trauma (Ghafoori et al., 2013; Tolin & Foa, 2008). In addition, gender did moderate the link between interpersonal trauma and PTSD, but not the link between non-interpersonal trauma and PTSD. When the traumatic experience was interpersonal in nature (medium or high betrayal), the link between the number of traumatic experiences and PTSD was significantly stronger for women than for men. The results therefore suggest that women are disproportionately affected by interpersonal trauma. These findings point to the importance of controlling for non-deployment-related trauma exposure and, in particular, interpersonal trauma in future research in order to accurately assess gender differences in PTSD.

### 4.1. Implications and future directions

Taken together, the findings provide valuable insights and indicate potential future directions for research on women who deploy and gender differences in PTSD following exposure to different types of trauma. Although women have a marginally higher probability of reporting PTSD following deployment-related trauma, they do not appear to be disproportionately at risk of developing deployment-related PTSD compared to men. Although additional research is required, the results suggest that exposure to deployment-related-traumas does not appear to pose an increased risk for women.

Controlling for non-deployment-related trauma did decrease gender differences in reported PTSD rates; however, marginally significant differences remained. One potential explanation for the greater prevalence of PTSD in women is that such differences pre-date any exposure to deployment-related trauma. To determine whether this is the case, it would be useful to establish whether any gender differences in PTSD symptoms exist among individuals with no traumatic experience. This was not possible in the current study because of the small number of respondents without deployment-related trauma and the non-exhaustive nature of the deployment-related trauma inventory.

The results of the current study provided some indication that exposure to non-deployment trauma contributed to gender differences in PTSD. In particular, the findings indicate that women are disproportionally affected by interpersonal trauma, suggesting United States special consideration should be given to these types of trauma in future research on gender differences in PTSD.

The finding that women appear to be disproportionately at risk of PTSD following interpersonal trauma also further indicates the importance of addressing interpersonal trauma exposure within the military. Recently, light has been shed on sexual misconduct and assault in the military context. A report by Statistics Canada (2016) indicated that female military personnel were four times more likely than male personnel to report being sexually assaulted in the past 12 months (women = 4.8%; men = 1.2%). In addition, 27.3% of women in the military report that they have been victims of sexual assault at some point during their military career (i.e. since joining the CAF) compared to only 3.8% of male personnel. In the United States, the Department of Defense released a report that indicated 4.3% of women on active duty compared to 0.5% of men on active duty reported experiencing sexual assault in 2016 (Department of Defense, 2017). Given the finding that women are at increased risk of developing PTSD when experiencing interpersonal trauma, it is important that military organizations continue to address sexual assault and unwanted touching in the military context to reduce their frequency of occurrence.

### 4.2. Limitations

Despite being based on a large sample, there were far fewer females compared to male respondents. While this is representative of the make-up of the larger military population it still resulted in some challenges. For example, there was a limited number of female respondents in some cells (e.g. more than four instances of deployment-related trauma), resulting in the need to collapse the trauma variables into categories to produce sufficiently reliable estimates. It was not possible to determine whether any gender differences in PTSD symptoms exist among individuals with no deployment-related traumatic experience, as very few respondents reported experiencing no deployment-related trauma. Had gender differences existed in this subgroup, it would have supported the view that gender differences in PTSD pre-date exposure to deployment-related trauma. It is worth noting that the models were run with the trauma variables as continuous factors and the results were consistent.

The proportionally small number of women was also linked to another limitation, the categorization of the trauma variables. As mentioned in the Method section, because of the small number of respondents for some of the open-ended response options (e.g. few women experienced more than four deployment trauma experiences), we chose to treat trauma exposure as categorical variables reflecting low, medium, and high exposure to traumatic events based on approximate tertiles of exposure. Although categorization of variables can often lead to a reduction in analytic power, a small number of respondents for some options may have resulted in unstable estimates.

In addition, we were unable to examine the differential effects of military sexual trauma compared to other sexual traumas. The structure of the questionnaire was such that the items could not distinguish between those with military sexual trauma *alone* and those with military sexual trauma *in conjunction with other sexual trauma*. In addition, limitations in the survey items precluded us from controlling for childhood trauma beyond a single item (i.e. beaten as a child by a caregiver). It may be valuable to account for these variables in future research.

This study examined deployment-related trauma as a broad category; however, there is evidence that the relationship between deployment-related trauma and mental health problems varies depending on the specific type of trauma. In one study of Canadian military personnel, specific combat events that were most strongly associated with PTSD were those that were uncommonly experienced, were unexpected, and could be interpreted as reflecting some violation of one’s morality (Watkins, Sudom, & Zamorski, 2016). Thus, it is possible that gender differences exist in the responses to different specific types of deployment-related trauma. While assessing this was beyond the scope of the present study, this could be explored in future research. A final limitation relates to the nature of the mental health outcome that was examined in the present study. The choice to focus on lifetime PTSD as opposed to past-year PTSD (or past-year subthreshold PTSD) primarily related to our desire to focus as much as possible on *incidence* as opposed to *persistence* of PTSD, which past-year outcomes also capture. The relationship of trauma exposure and gender with incidence may differ for persistence, adding unwanted heterogeneity to the analysis. The greater statistical power afforded by a higher outcome density of lifetime compared to past-year disorders was also a consideration. It is important, however, to point out two key disadvantages of our choice of outcome. First, PTSD is only one of many possible mental health problems that may follow exposure to trauma (Sareen et al., 2007), although trauma exposure tends to have the strongest (and, hence, most readily detectable) relationship with PTSD (Boulos & Zamorski, 2013). Future work could explore trauma and gender interactions for other outcomes. Second, the prolonged recall period for lifetime PTSD increases the possibility that some of the traumas we measured had *followed* incident PTSD (hence, could not have contributed to it). Future analyses with better assessment of the temporality of trauma and onset PTSD are needed to address this limitation.

### 4.3. Conclusion

Although accounting for non-deployment-related trauma in the present analyses did not fully dissipate the gender differences in PTSD following deployment-related trauma, such differences were greatly reduced, suggesting these were at least partly attributable to non-deployment-related trauma. As gender did not moderate the link between deployment-related trauma and PTSD, it appears that traumatic events experienced while on deployment do not disproportionately affect women. In addition, other findings support betrayal theory, indicating that the association between interpersonal trauma and PTSD was stronger among female than among male service members. This finding suggests that interpersonal trauma should be a control variable when examining gender in relation to mental health outcomes. It also stresses the importance of reducing interpersonal trauma exposure within the military. In future research on gender differences in mental health outcomes, it will be important to consider the contribution of interpersonal trauma. Moreover, given the various impacts that exposure to trauma may have on mental health (Sareen et al., 2007), it may be worth conducting additional research to examine gender differences in the association between combat and a wider range of mental health outcomes.
